# Supplementary material for: From sequence to enzyme mechanism using multi-label machine learning
Source: BMC Bioinformatics. 2014 May 19;15:150. doi: 10.1186/1471-2105-15-150 (PMC4229970; doi:10.1186/1471-2105-15-150)
Supplement: Additional file 2 — Java code of ml2db. Additional file ml2db_code.tar.gz contains the Java source code to run the multi-label machine learning experiments and save the results to database. The code’s Javadoc is included. [file 1471-2105-15-150-S2.zip › additional file 2/ml2db/ml2db/doc/main/ml2db/mulan/mulanarff/MulanArff.html]

MulanArff


JavaScript is disabled on your browser.


- Overview
- Package
- Class
- Use
- Tree
- Deprecated
- Index
- Help

- Prev Class
- Next Class

- Frames
- No Frames

- All Classes

- Summary:
- Nested |
- Field |
- Constr |
- Method

- Detail:
- Field |
- Constr |
- Method


main.ml2db.mulan.mulanarff

## Class MulanArff

- java.lang.Object
- - uk.ac.standrews.utils.main.Initialised
  - - main.ml2db.weka.Arff
    - - main.ml2db.mulan.mulanarff.MulanArff

- All Implemented Interfaces:
  :   uk.ac.standrews.utils.main.Initialisable

  Direct Known Subclasses:
  :   AttFiltArff

  ---

    

  ```
  public class MulanArff
  extends Arff
  ```

  Generates an Attribute Relation Format File (ARFF) in Mulan format (multiple
  class labels per instance). The class attributes must be in a separate Mulan
  XML file (either flat or hierarchical) to distinguish them from the normal
  attributes.
  Call structure: the MulanArffGenerator creates a DataSetManager which checks
  the input (DataSetChecker), loads the data (MulanDataSetDbLoader), creates
  the instances (MulanDataSetGenerator) and writes them as ARFF file
  (DataSetWriter).
  beware! the class attributes must always come alphabetically before the
  feature attributes. It's a bug, I know, never came round to sort it. It only
  affects predictions emission. Append an 'a' to class attributes and a 'z' to
  feature attributes...

  Version:
  :   10 May 2010

  Author:
  :   Luna De Ferrari ldf(at)st-andrews.ac.uk

- - ### Field Summary

    Fields

    | Modifier and Type | Field and Description |
    | --- | --- |
    | `boolean` | `m_massiveFile` if plenty of memory available, also load each instance in weka instances, otherwise only write them to file one by one |

    - ### Fields inherited from class uk.ac.standrews.utils.main.Initialised

      `m_initialisationIsCorrect`
  - ### Constructor Summary

    Constructors

    Constructor and Description

    `MulanArff(java.lang.String dataQueriesProps,
    DbManagers dbMan)`

    `MulanArff(java.lang.String dataQueriesProps,
    DbManagers dbMan,
    boolean massive)`
  - ### Method Summary

    Methods

    | Modifier and Type | Method and Description |
    | --- | --- |
    | `java.lang.String` | `deriveXmlPath()` |
    | `java.lang.String` | `generateXmlFile(java.util.TreeSet<java.lang.String> classLabels)` Generate a hierarchical XML files of the classes |
    | `MulanDataSetManager` | `getDataSetManager()` |
    | `weka.core.Instances` | `getInstances()` |
    | `java.util.Vector<java.lang.String>` | `getInstancesNames()` |
    | `static java.lang.String` | `getXMLpathFromArffPath(java.lang.String arffPath)` Generates the xml file path: same name+path as .arff file, but .xml termination |
    | `boolean` | `isMassiveFile()` |
    | `static void` | `main(java.lang.String[] args)` |
    | `static int[]` | `run(java.lang.String[] arffPaths, DbManagers dbMan, boolean massive)` Generates a number of ARFF files (the database the records are written is the same for all ARFF files) |
    | `static int` | `run(java.lang.String arffPropsPath, DbManagers dbMan, boolean massive)` Generates the ARFF (and XML) files and returns the identifier of the arff record in the database |
    | `void` | `setDataSetManager()` |

    - ### Methods inherited from class main.ml2db.weka.Arff

      `checkAndInitialise, fillArff, generateAndWriteArffFile, getArffPath, getArffRecordId, getDataArffProperties, getDataDbManager, getDataPropsFilePath, getDbConn, getDbManagers, getHeaderString, getInputDbReader, getInstancesString, getResultsDbManager, setArffRecordId, setup, writeArff`
    - ### Methods inherited from class uk.ac.standrews.utils.main.Initialised

      `initialisationIsCorrect, setInitialisation, setInitialisationIsCorrect, setInitialisationNotCorrect`
    - ### Methods inherited from class java.lang.Object

      `equals, getClass, hashCode, notify, notifyAll, toString, wait, wait, wait`

- - ### Field Detail


    - #### m\_massiveFile

      ```
      public boolean m_massiveFile
      ```

      if plenty of memory available, also load each instance in weka instances,
      otherwise only write them to file one by one
  - ### Constructor Detail


    - #### MulanArff

      ```
      public MulanArff(java.lang.String dataQueriesProps,
               DbManagers dbMan)
      ```


    - #### MulanArff

      ```
      public MulanArff(java.lang.String dataQueriesProps,
               DbManagers dbMan,
               boolean massive)
      ```
  - ### Method Detail


    - #### generateXmlFile

      ```
      public java.lang.String generateXmlFile(java.util.TreeSet<java.lang.String> classLabels)
      ```

      Generate a hierarchical XML files of the classes


    - #### getDataSetManager

      ```
      public MulanDataSetManager getDataSetManager()
      ```

      **Overrides:**
      :   `getDataSetManager` in class `Arff`


    - #### getInstances

      ```
      public weka.core.Instances getInstances()
      ```

      **Overrides:**
      :   `getInstances` in class `Arff`


    - #### getInstancesNames

      ```
      public java.util.Vector<java.lang.String> getInstancesNames()
      ```


    - #### deriveXmlPath

      ```
      public java.lang.String deriveXmlPath()
      ```


    - #### isMassiveFile

      ```
      public boolean isMassiveFile()
      ```


    - #### setDataSetManager

      ```
      public void setDataSetManager()
      ```

      **Overrides:**
      :   `setDataSetManager` in class `Arff`


    - #### getXMLpathFromArffPath

      ```
      public static java.lang.String getXMLpathFromArffPath(java.lang.String arffPath)
      ```

      Generates the xml file path: same name+path as .arff file, but .xml
      termination

      Parameters:
      :   `arffPath` - the path to the arff file

      Returns:
      :   the same filename+directorypath of the arff file but with the
          termination .xml instead of .arff


    - #### main

      ```
      public static void main(java.lang.String[] args)
      ```

      Parameters:
      :   `args` -


    - #### run

      ```
      public static int run(java.lang.String arffPropsPath,
            DbManagers dbMan,
            boolean massive)
      ```

      Generates the ARFF (and XML) files and returns the identifier of the arff
      record in the database

      Parameters:
      :   `arffPropsPath` - the path of the ARFF properties file (queries that define the
          data to be included)
      :   `machineLearningProps` - the database connection to the database containing the arff
          file table, to save the arff file details.

      Returns:
      :   the identifier of the generated ARFF file in the database table


    - #### run

      ```
      public static int[] run(java.lang.String[] arffPaths,
              DbManagers dbMan,
              boolean massive)
      ```

      Generates a number of ARFF files (the database the records are written is
      the same for all ARFF files)

      Parameters:
      :   `arffPaths` - a list of arff properties files (containing the data queries)
      :   `machineLearningProps` - the database connection properties file to write the arff
          records too
      :   `whether` - the arff file is so big it must be written on the fly

      Returns:
      :   a list of arff records identifiers in the database arff table


- Overview
- Package
- Class
- Use
- Tree
- Deprecated
- Index
- Help

- Prev Class
- Next Class

- Frames
- No Frames

- All Classes

- Summary:
- Nested |
- Field |
- Constr |
- Method

- Detail:
- Field |
- Constr |
- Method
